# High-Throughput Sequencing, Characterization and Detection of New and Conserved Cucumber miRNAs

**DOI:** 10.1371/journal.pone.0019523

**Published:** 2011-05-16

**Authors:** Germán Martínez, Javier Forment, Cesar Llave, Vicente Pallás, Gustavo Gómez

**Affiliations:** 1 Instituto de Biología Molecular y Celular de Plantas (IBMCP), Consejo Superior Investigaciones Científicas (CSIC) - Universidad Politécnica de Valencia (UPV), Valencia, Spain; 2 Centro de Investigaciones Biológicas (CIB), Consejo Superior Investigaciones Científicas (CSIC), Madrid, Spain; St. Georges University of London, United Kingdom

## Abstract

Micro RNAS (miRNAs) are a class of endogenous small non coding RNAs involved in the post-transcriptional regulation of gene expression. In plants, a great number of conserved and specific miRNAs, mainly arising from model species, have been identified to date. However less is known about the diversity of these regulatory RNAs in vegetal species with agricultural and/or horticultural importance.

Here we report a combined approach of bioinformatics prediction, high-throughput sequencing data and molecular methods to analyze miRNAs populations in cucumber (*Cucumis sativus*) plants. A set of 19 conserved and 6 known but non-conserved miRNA families were found in our cucumber small RNA dataset. We also identified 7 (3 with their miRNA* strand) not previously described miRNAs, candidates to be cucumber-specific. To validate their description these new *C. sativus* miRNAs were detected by northern blot hybridization. Additionally, potential targets for most conserved and new miRNAs were identified in cucumber genome.

In summary, in this study we have identified, by first time, conserved, known non-conserved and new miRNAs arising from an agronomically important species such as *C. sativus*. The detection of this complex population of regulatory small RNAs suggests that similarly to that observe in other plant species, cucumber miRNAs may possibly play an important role in diverse biological and metabolic processes.

## Introduction

The discovery of MicroRNAs (miRNAs) is a milestone in the development of modern biology. MiRNAs are a class of endogenous ∼21 nucleotide (nt) small non-coding RNA (sncRNA) presents in both plants and animals. In plants, mature miRNAs are processed by the DICER-LIKE 1 (DCL1) RNase III-like protein from a longer nuclear-localized RNA transcript that forms a fold-back stem-loop structure of partially complementary double-stranded RNA (dsRNA). miRNAs regulate gene expression in a sequence-specific manner via degradation of target mRNAs or inhibition of protein translation [1], [2]. Increasing evidence indicates that miRNAs, together with other non-coding RNA families, play major roles in plant development and response to biotic and abiotic stress [3]. Initially, miRNAs were identified by conventional low-scale sequencing of cDNA clones from small RNA libraries prepared from different plant sources.

Subsequent studies showed that a significant subset of plant miRNAs are strictly conserved across different species within the plant kingdom [4]. Alternatively it was also reported that a smaller proportion of miRNAs, often expressed at low levels, were absent in diverse species suggesting that they could have evolved more recently [5]. As a result, non-conserved miRNAs that are rarely retrieved from low-scale sequencing experiments remain poorly characterized in most plant species. However, the recent irruption of high-throughput sequencing technologies has allowed deeper sampling of the small RNA populations enabling the identification of a substantial number of non-conserved miRNAs in diverse vegetal-species [6]–[11].

At least, two criteria involving expression and biogenesis parameters have been adopted for miRNA annotation [12], [13]. A primary criterion establishes that candidate miRNAs should be supported by in vivo detection or cloning of the ∼21-nucleotide miRNA/miRNA* duplex that arise from the stem of a single-stranded, stem-loop precursor [13]. Second, the satisfaction of one or more of the follow ancillary criteria can increase the confidence of a miRNA annotation: (i) the fold-back structure should be phylogenetically conserved; (ii) the precursor should be shown to accumulate in organisms with impaired Dicer function; and (iii) the expression of the predicted miRNAs should be validated by *Northern blot* hybridization and/or PCR amplification [12]. Inspired in these annotation criteria, to date, more than 2,500 conserved and recently evolved species-specific miRNAs have been deposited in the miRNA Registry Database (miRBase Release 16.0, September 2010; http://microrna.sanger.ac.uk/).

Cucumber (*Cucumis sativus L.*) is among the 20 most important vegetable crops worldwide (http://faostat.fao.org), and it has been proposed as a model plant for vascular and sexual development studies [14], [15]. However, despite of its agronomic and biological importance and the recent publication of the complete genome [16], miRNAs in cucumber have not been reported yet. To take advantage of the recently published sequence data of endogenous small-RNAs recovered from cucumber [17], here we combined bioinformatics with high-throughput sequencing data to provide the first inventory of cucumber miRNAs population. We identified 25 previously described plant-miRNA families as well as 7 additional unknown miRNAs. The accumulation of a representative set of conserved and new cucumber miRNAs was experimentally validated by northern blot assays, using non-isotopic miRNA-specific probes.

## Results

### Analysis of *C. sativus* sRNA population

Starting from a cDNA library of short sRNAs from *C. sativus* containing 209,331 high quality sequences [17] we selected 38,747 non-redundant reads recovered from cucumber leaves with lengths of 18 to 30 nts to analyze the general profile of cucumber sRNAs. This data set was analyzed using the online University of East Anglia plant sRNA toolkit [18].

**Figure 1:**
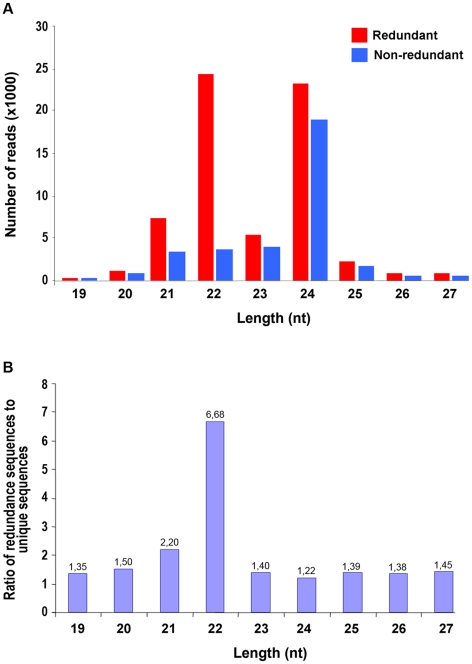
Size distribution of small RNAs (sRNAs) sequences. A) The number of redundant (red bars) and non-redundant (blue bars) sequences recovered from the *C. sativus* sRNA library is expressed as thousand unitis. B) Redundancy ratio for cucumber sRNAs.

Size distribution of unique sequences is summarized in Fig. 1. The majority of the reads (82.62%) were in the range of 20 to 24 nt in length, with 24 nts (51.15%) being the most represented class of non-redundant species followed by 23 (10.40%), 22 (9.81%) and 21 nt (9.08%). This result was consistent with that previously reported for other plant species such as *Arabidopsis*, *Medicago truncatula*, *Oriza sativa*, *Populus spp.* and *Citrus trifoliate* where 24-nt sRNAs dominate the sRNA transcriptome [6]–[9], [11], [19]. The analysis of the average abundance of total cucumber sRNAs measured by the ratio of raw and unique sequences revealed that the 24-nt class exhibited high sequence diversity consistent with the widespread origins of sRNAs of this size along plant genomes. Surprisingly, the highest level of redundancy (6.68) was found within the 22 nt sRNA subset (Fig. 1, blue bars).This atypical situation, draws a parallel with the relatively high number of redundant 22-nt sequences obtained from *C. trifoliate* by Solexa (Illumina) high-throughput sequencing [11].

### Identifying known miRNAs in *C. sativus*

Known miRNAs comprise both conserved and non-conserved miRNA species. In order to identify known miRNAs in our sequenced set of cucumber sRNAs, we compared the sequences recovered from our library with the repository of mature plant-miRNAs in miRBase (Release 16.0, September 2010; http://microrna.sanger.ac.uk) using the miRProf tool of the sRNA tool kit. Based on sequence homology our analysis revealed the presence of 25 known plant-miRNA families in our dataset (Table 1). The identified known-miRNAs corresponded to 19 conserved (miR156, miR159, miR160, miR164, miR165, miR166, mi167, miR168, miR169, miR171, miR172, miR319, miR390, miR393, miR396, miR397, miR398, miR398 and miR408) and 6 known but non-conserved (miR170, miR827, miR858, miR894, miR1030 and miR2950) miRNA families. As expected, most of the miRNAs identified in cucumber were highly conserved in diverse plant species [20], suggesting that the ancient regulatory pathways mediated by evolutionary conserved miRNAs are also functional in cucumber plants. The stable expression level of a selected set of conserved miRNAs (miR159, miR167 and miR168) in diverse plant sectors across successive growing phases (Figure S1) strongly supports this idea.

**Table 1:** Known micro RNAs (miRNAs) recovered from our *C. sativus* sRNA library.

miRNA family	Size range	5′ end	miRNA*	Homology by specie					
				A.thaliana	O.sativa	P.trichocharpa	R.comunis	M.truncatula	V.vinifera
**Conserved**									
**miR156**	20–23	U (68%)	Yes	Yes	Yes	Yes	Yes	Yes	Yes
**miR159**	19–23	U (77%)	No	Yes	Yes	Yes	Yes	Yes	Yes
**miR160**	20–21	U (100%)	Yes	Yes	Yes	Yes	Yes	Yes	Yes
**miR164**	20–21	U (67%)	No	Yes	Yes	Yes	Yes	Yes	Yes
**miR165**	21	U (100%)	No	Yes	Yes	No	No	No	No
**miR166**	21–22	U (100%)	Yes	Yes	Yes	Yes	Yes	Yes	Yes
**miR167**	21–23	U (87%)	No	Yes	Yes	Yes	Yes	Yes	Yes
**miR168**	21–22	U (75%)	Yes	Yes	No	Yes	Yes	Yes	Yes
**miR169**	21–23	U (75%)	Yes	Yes	No	Yes	No	No	Yes
**miR171**	19–23	U (65%)	Yes	Yes	Yes	Yes	Yes	Yes	Yes
**miR172**	19–21	A/G (50%)	Yes	Yes	Yes	Yes	Yes	Yes	Yes
**miR319**	20–21	U (100%)	No	Yes	No	Yes	Yes	Yes	Yes
**miR390**	19–21	A (75%)	Yes	Yes	Yes	Yes	Yes	Yes	Yes
**miR393**	21–23	U (100%)	No	Yes	Yes	Yes	Yes	Yes	Yes
**miR396**	21–22	U (100%)	Yes	Yes	Yes	Yes	Yes	Yes	Yes
**miR397**	21	C (50%)	No	Yes	Yes	Yes	Yes	No	Yes
**miR398**	20–21	U (100%)	No	Yes	Yes	Yes	Yes	Yes	Yes
**miR399**	21	U (100%)	No	Yes	No	No	No	No	No
**miR408**	21–22	U (66%)	No	Yes	Yes	Yes	Yes	Yes	Yes
**Non-conserved**									
**miR170**	21–22	U (100%)	No	No	No	No	No	No	Yes
**miR827**	22	U (100%)	No	No	No	Yes	No	No	No
**miR858**	22	U (100%)	No	No	No	Yes	Yes	No	No
**miR894**	20–21	G/C(50%)	No	No	No	Yes	No	No	No
**miR1030**	19	C (100%)	No	No	No	Yes	No	No	No
**miR2950**	21–22	U (100%)	Yes	No	No	No	No	No	Yes

*The column shows the reads encompassing the defined miRNA sequence ±2 nts.

**Figure 2:**
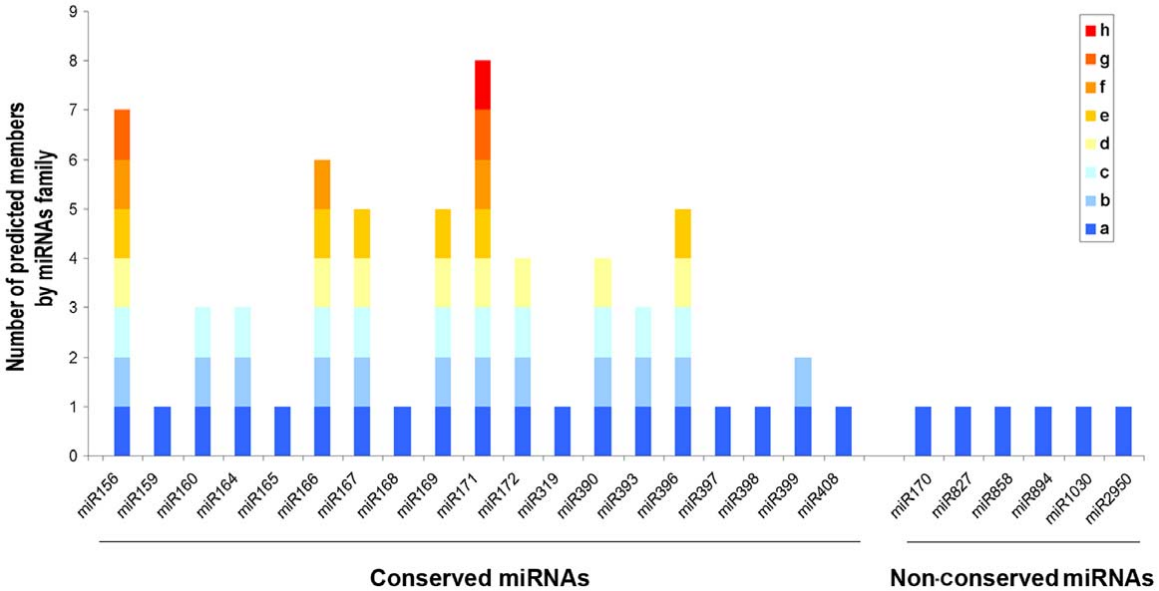
Complexity of the known miRNA families identified in *C. sativus*. Graphic representation of the different members of each conserved and non conserved miRNA family found in cucumber by sequencing and miRNA precursors prediction (miRCat).

Next, potential miRNA precursors of conserved cucumber miRNAs were identified by Blastn against the cucumber transcript database currently available at http://www.phytozome.net/cucumber.php. Secondary structure analysis revealed that pre-miRNAs holding characteristic secondary structures were predicted for 18 of the 25 miRNA families identified by sequence homology (Table S1). This finding added robustness to our data obtained by high throughput sequencing. The predicted hairpins have a free energy ranging from −37.76 to −100.91 ▵G and a predicted length ranging from 85 to 283 nts. The majority (12 out of 18) of the conserved miRNA families were composed by more than one member originating from different genomic loci, whereas the totality of the non-conserved known miRNAs arises from a single locus (Fig. 2).

**Figure 3:**
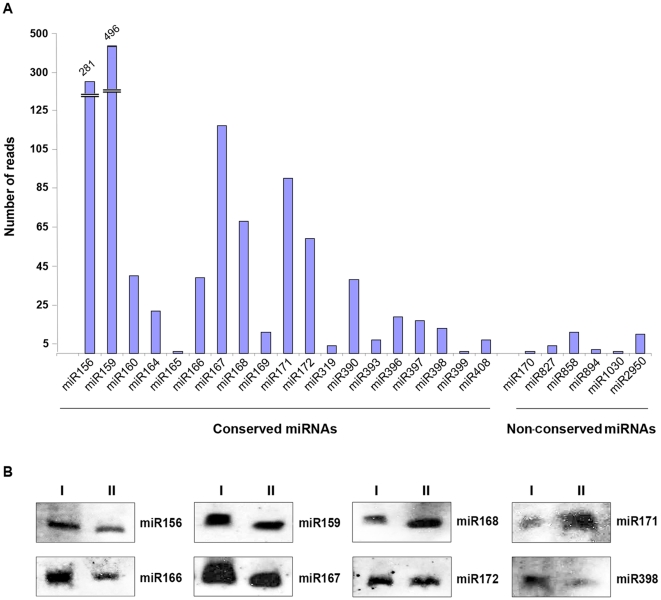
Analysis of known miRNAs found in cucumber. A) Graphic showing the number of sequences for each (conserved and non-conserved) miRNA family. The values on the *y*-axis represent the abundance of reads in the library. B) Detection of selected known cucumber miRNAs by *Northern blot* assays using non isotopic probes. I and II indicate plants maintained at 25°C and 30°C, respectively, resembling the plant growing conditions used for sRNAs library generation.

Since high-throughput sRNA sequencing provides the opportunity for quantitative profiling of sRNA populations, sequencing frequencies in our sequenced collection were employed as an estimation of miRNA abundance. Counting of redundant sRNA reads revealed that 14 out of 19 conserved miRNAs were represented with more than 10 reads in the cucumber data set; only miR165, miR399, miR319, miR393 and miR408 had less than ten reads. By contrast, less than 10 reads were counted for most known, non-conserved miRNA families; exceptions were miR858 and 2950 with more than 10 reads in the sequenced set (Fig. 3A). Next, to confirm their expression in cucumber, a representative group of conserved miRNAs were analyzed by Northern blot analysis. As observed in the Fig. 3B, the totality of the tested miRNA were readily detected in the leaves of cucumber plants maintained at two different temperatures (see material and methods) using non-isotopic hybridization techniques, suggesting that they were abundant as predicted by their respective sequencing frequencies.

### Identifying new potential miRNAs in *C. sativus*

**Figure 4:**
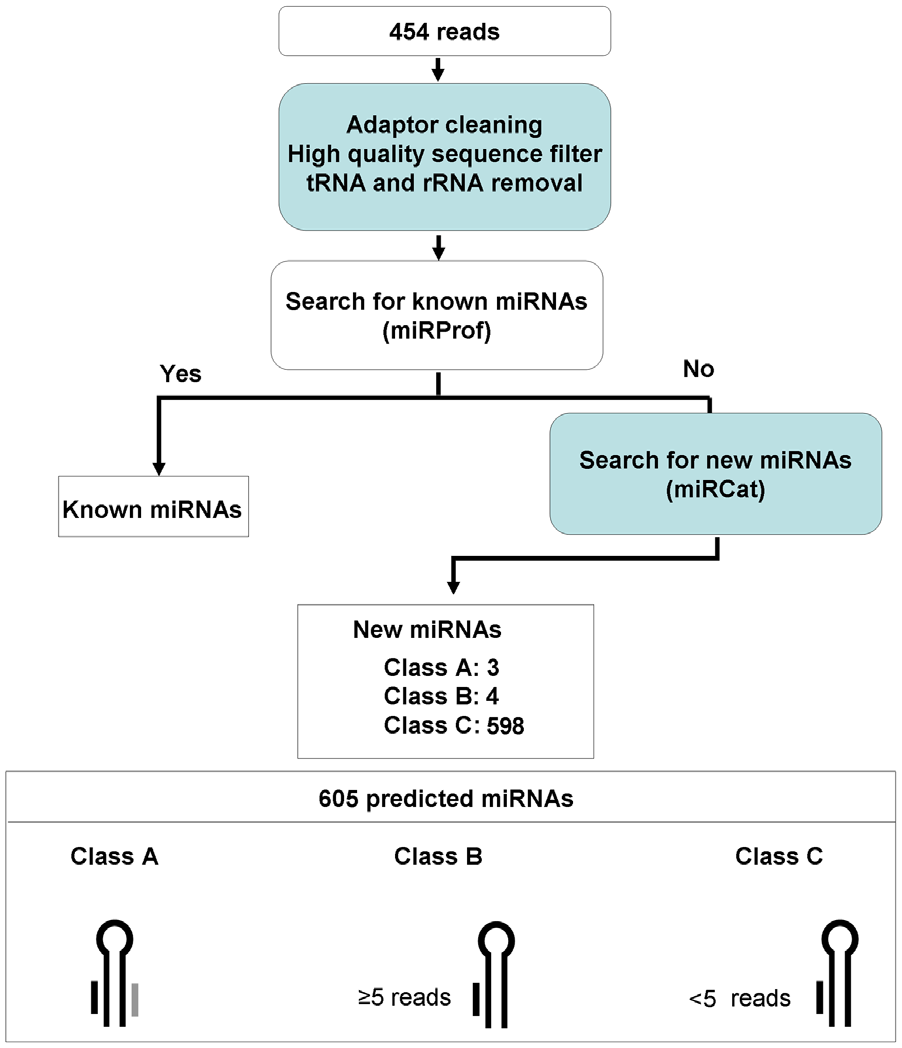
Flowchart for the identification of new miRNAs on *C. sativus*.

To discover additional non-conserved, cucumber-specific miRNA candidates within our sequenced set, unique small RNA species were aligned against the *C. sativus* genome sequence in order to identify loci that may serve as putative precursors for miRNA. A total of 605 potential candidate new miRNAs were selected for further analysis and classified into three arbitrary categories. Class ***A*** comprised sRNAs that had their star counterpart sequenced; class ***B*** contained sRNAs that had been sequenced more than 5 times but were not supported by star counterparts; class ***C*** included rare sRNAs represented by less than five reads in the sequenced set (Fig. 4). Besides, sRNAs that hit the cucumber genome at more than 50 genomic loci were regarded as likely repeat-associated siRNAs and excluded from the analysis.

**Figure 5:**
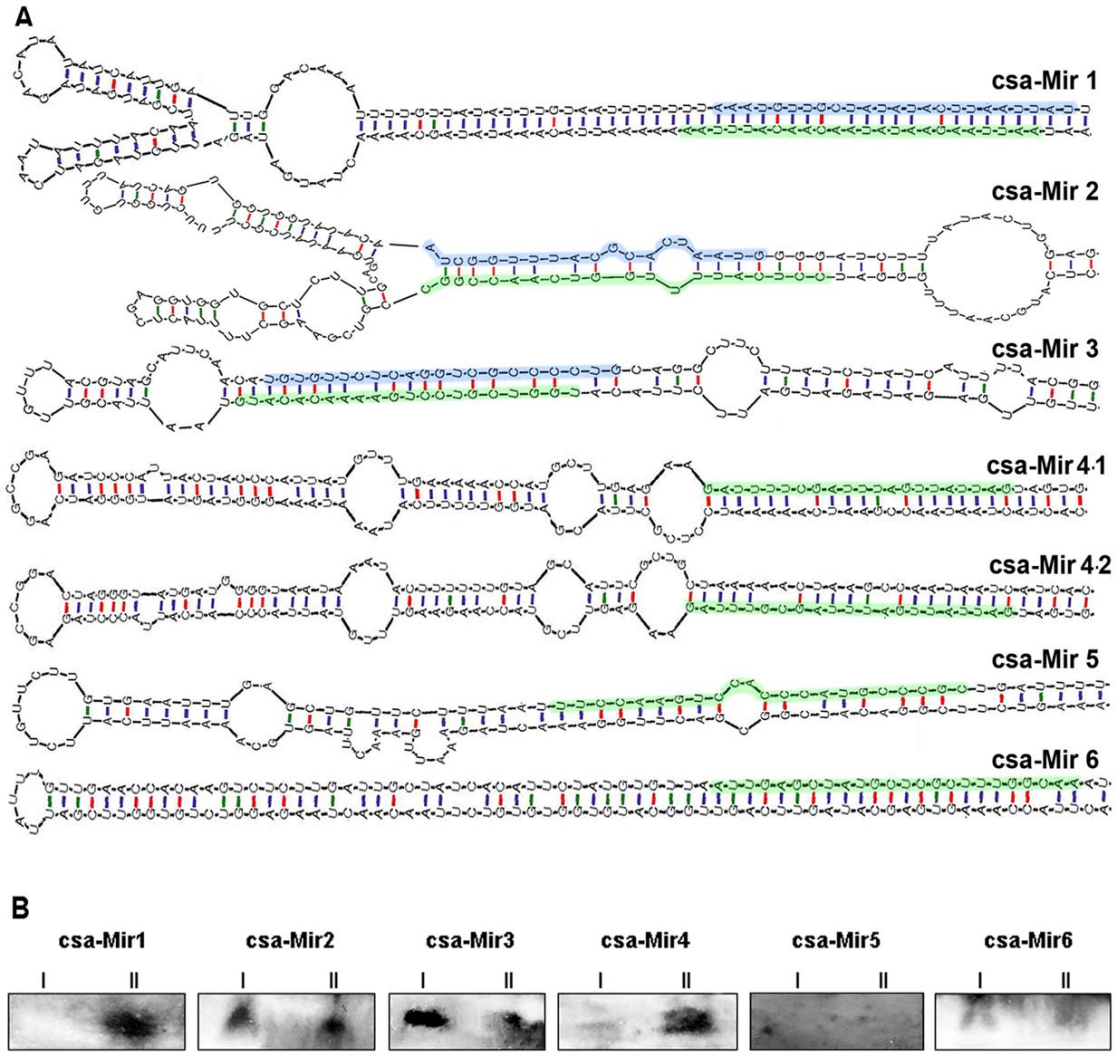
Bioinformatics prediction and Northern blot detection of new *C. sativus* miRNAs. A) Primary transcripts containing the predicted stem-loop structures precursor of new cucumber miRNAs. The mature miRNAs identified in cucumber sRNAs library are highlighted (green for miRNAs and blue for miRNAs*). The size of the precursors may be slightly longer than represented. B) Low molecular-weight enriched-RNA was extracted from cucumber leaves, electrophoresed and transferred to nylon membranes. The novel cucumber miRNAs were detected by *Northern blot* assays using non isotopic probes. I and II indicate plants maintained at 25°C and 30°C, respectively, resembling the plant growing conditions used for sRNAs library generation.

Our analysis revealed 7 possible miRNA sequences deriving from genomic loci with intramolecular folding capacity that resembled the characteristic and predictable local hairpin structures of miRNA precursors (Fig. 5A and Table 2). Three of these candidate miRNAs (csa-miR1, csa-miR2 and csa-miR3) belonged to class ***A***, and were considered as authentic miRNAs as they satisfactory fulfilled the expression and biogenesis criteria (sequencing of miRNA* strand) for miRNA annotation. The remaining four sequences belonged to class ***B*** (csa-miR4.1, csa-miR4.2, csa-miR5 and csa-miR6) and were considered as possible candidates because they apparently derived from stem-loop structures and were relatively abundant, but miRNA* species were not sequenced. In accordance with that reported for other plant miRNAs, these newly identified miRNAs derive from predicted hairpin structures ranging from 134 to 230 nts long. The minimum free energies of these pre-miRNA hairpin structures rang from −42.00 to −97.90 ▵G. The described cucumber miRNAs were potentially generated from 7 different loci (Table 2). The new miRNAs included in the class ***A*** (csa-miR1, csa-miR2 and csa-miR3) and the candidates (class ***B***) miRNAs (csa-miR5 and csa-miR6) each arose from a single locus, in good agreement with most species-specific miRNAs detected in other plant species. The candidate csa-miR4 is predicted to be produced from two loci. As expected, none of the new described cucumber-miRNAs was found to be clustered (data not shown), since this organization pattern (common in animals) is infrequent in plants, with the exception of some species like soybean, grapevine or tobacco [10], [21], [22]. To validate their prediction and examine their expression we analyzed leaves of cucumber plants maintained at 25°C and 30°C temperatures both resembling the plant growing conditions used during the sRNAs library generation. The new cucumber miRNAs were subjected to sRNA hybridization analysis against non-isotopic riboprobes (Fig. 5B). All the Class ***A*** cucumber-specific miRNAs (csa-miR1, csa-miR2 and csa-miR3) and 2 Class ***B*** candidate-miRNAs (csa-miR4 and csa-miR6) were detected by Northern blot assays, providing additional robustness to our prediction. The candidate csa-miR5, that shows a low intensity signal at high exposition times, was considered as undetected.

**Table 2:** Novel miRNAs found in *Cucumis sativus*.

csa-MIR	Location	Orientation	Abundance	Sequence	Length (nt)	Hairpin Length (nt)	Minimum Free Energy	Class	miRNA*
**1**	scaffold03050:42052.42075	+	1	AATTAAGAATATAACAACATTTAA	24	230	−67.60	A	AAATGTTGCTATATACTTAATTAT(1)
**2**	scaffold04026:144.163	+	1	CCTCATTTTGGTCAACCGGC	20	155	−49.69	A	ATCGGTTTACGCACTAATG(1)
**3**	scaffold03577:144799.144819	+	10	TGGTCGTCCTGAAAACACATG	21	134	−42.00	A	TGTGTTCTCAGGTCGCCCCTG(8)
**4_1**	scaffold01087:76.96	+	6	GAAGGCTGTGATGATTATTGA	21	223	−97.90	B	NO
**4_2**	scaffold04146:100.120	+	6	GAAGGCTGTGATGATTATTGA	21	225	−94.50	B	NO
**5**	scaffold02633:580263.580284	−	10	TTCCAAGTCCACCCATGCCCGC	22	185	−72.10	B	NO
**6**	scaffold01374:173181.173204	−	59	ATTGAGCTATGCTCGCTTTGGCAA	24	196	−91.00	B	NO

*Location of the miRNA sequence on the cucumber genome, indicating the scaffold from which the miRNA derives and the start and end position.

### Prediction of miRNA targets in *C. sativus*

With the aim to better understand the biological functions of the newly identified as well as known (conserved and non-conserved) *C. sativus* miRNAs, we searched for putative target genes by using the plant target prediction tool of the sRNA tools (http://srna-tools.cmp.uea.ac.uk/plant/cgi-bin/srna-tools) and the *C. sativus* transcript database (http://www.phytozome.net/cucumber.php) as the reference set. As a result, we found putative target genes for 1 out of 3 new cucumber miRNAs and for 2 out of 3 candidate cucumber miRNAs (Table 3). Since, the recently sequenced cucumber genome has not been functionally annotated yet, we searched for homologous proteins for these cucumber transcripts in the NCBI protein-database (www.ncbi.nlm.nih.gov) in order to infer the biological functions of their predicted targets. The known proteins, homologous to the putative targets genes, appear to be involved in a wide variety of biological processes (Table 3). For example, the predicted csa-miR2 target was a gene coding a tetratricopeptide repeat-containing protein, a kind of protein involved in protein-protein and/or protein-RNA interactions. Candidate csa-miR4/1 and 4/2 had two predicted targets, a nodulin similar to MtN21 family protein which are related to vascular tissue development, nodule formation and transport functions, and a clathrin binding protein involved in plant endocytosis. Csa-miR5 targets are R-FOM2, a resistance gene to *Fussarium oxysporum* f.sp. melonis from *C. melo*, and a r2r3-myb transcription factor which plays important roles in the regulation of many secondary metabolites at the transcriptional level. To obtain more complete information, we blasted against the cucumber draft genome the potential cucumber specific miRNAs with unpredicted target in the coding transcriptome. This analysis revealed that csa-Mir1 and csa-Mir6 possess highly homology (>91%) with diverse RNA regions not identified as protein coding (data no shown). No homologous region (except the corresponding to miRNA precursor) was identified in the cucumber genome for csa-Mir3.

**Table 3:** Predicted targets of new cucumber miRNAs.

miRNA	C. sativus target gene accession	start-end position of target	Target translation	Specie
**1**	No target found			
**2**	Cucsa.328090.2	147–166	Tetratricopeptide repeat (TPR)-containing protein (AT1G77230)	A.thaliana
**3**	No target found			
**4_1**	Cucsa.250100.1	860–876	Nodulin MtN21 family protein (AT5G64700)	A.thaliana
**4_1**	Cucsa.395290.1	1435–1448	Clathrin binding protein (At5g11710)	A.thaliana
**4_2**	Cucsa.250100.1	860–876	Nodulin MtN21 family protein (AT5G64700)	A.thaliana
**4_2**	Cucsa.395290.1	1435–1448	Clathrin binding protein (At5g11710)	A.thaliana
**5**	Cucsa.251930.1	592–613	R-FOM-2 gene	C.melo
**5**	Cucsa.368410.1	733–754	r2r3-myb transcription factor, putative	R.communis
**6**	No target found			

Next, we explored the *C. sativus* transcript database searching for predicted target genes of the previously known cucumber miRNAs described in this study. Putative targets were found in the cucumber transcriptome for the majority of these conserved and non-conserved known miRNAs (Table 4). Additionally, we compared for a representative set of conserved cucumber miRNAs, the target genes predicted in cucumber with the previously reported targets. As expected, all of these miRNAs share putative target genes with their homologous miRNAs in other plant-species (data not shown), reinforcing the idea that conserved plant-miRNAs are involved in essentials biological processes.

**Table 4:** Predicted targets of conserved and non-conserved cucumber miRNAs.

miRNA	Targeted Genes	Targeted Protein
**Conserved**		
**miR156**	Cucsa.054050.1, Cucsa.113410.2, Cucsa.157230.1, Cucsa.198630.1, Cucsa.242790.1, Cucsa.392170.1, Cucsa.349620.1, Cucsa.259800.1	Squamosa promoter binding protein
	Cucsa.291270.1	Cationic amino acid transporter
	Cucsa.303890.4	Short-chain dehydrogenase
	Cucsa.350960.1, Cucsa.278470.1	Unknown
**miR159**	Cucsa.094180.1, Cucsa.121070.1, Cucsa.148580.1, Cucsa.364400.1, Cucsa.094180.1, Cucsa.121070.1, Cucsa.364400.1, Cucsa.148580.1	R2R3-MYB transcription factor
	Cucsa.047860.3, Cucsa.111880.2	TCP transcription factor
	Cucsa.273670.1	BRASSINAZOLE-RESISTANT 2 protein
	Cucsa.283050.1	ARIADNE-like protein
	Cucsa.283050.1	pre-miRNA 395e
	Cucsa.273440.1	Hypothetical
	Cucsa.302420.2	Unknown
**miR160**	Cucsa.308310.1	miR160c gene
**miR164**	Cucsa.040380.1	NAC transcription factor
	Cucsa.122890.1	CUC transcription factor
	Cucsa.032500.1, Cucsa.112890.1, Cucsa.158180.2, Cucsa.197830.1, Cucsa.338700.3	NAM transcription factor
**miR165**	Cucsa.048220.1	PHB transcription factor
	Cucsa.167110.5, Cucsa.385410.3, Cucsa.386080.9	Class III HD-Zip protein
**miR166**	Cucsa.341260.1	PRA-7 protein
**miR167**	Cucsa.047990.2, Cucsa.152170.3, Cucsa.173450.1	Auxin response factor
	Cucsa.097070.2	Magnesium/proton exchanger
	Cucsa.139150.1	CDC6
	Cucsa.160570.1	MYB transcription factor
**miR168**	No target found	
**miR169**	Cucsa.055300.1	Tubulin gamma complex-associated protein
**miR171**	Cucsa.120600.1, Cucsa.201250.1	Scarecrow-like transcription factor
	Cucsa.320850.2	GRAS transcription factor
**miR172**	Cucsa.065640.1, Cucsa.102550.1, Cucsa.165940.1, Cucsa.307230.1	Apetala 2 transcription factor
	Cucsa.385890.1	pre-miRNA 172a
**miR319**	Cucsa.283050.1	miR395e promary transcript
	Cucsa.302420.2	Zinc finger protein
	Cucsa.364400.1	MYB transcription factor
**miR390**	Cucsa.089280.1	ZEITLUPE protein
	Cucsa.164200.1	Lucine-rich repeat receptor-like protein
**miR393**	Cucsa.156100.1	Auxin signaling f-box 2
	Cucsa.196530.1	Dead box ATP-dependent RNA helicase
**miR396**	No target found	
**miR397**	Cucsa.066920.1, Cucsa.067060.1, Cucsa.124400.1, Cucsa.146870.1, Cucsa.175530.1, Cucsa.176130.1	Laccase
	Cucsa.266970.1	Ubiquitin
**miR398**	No target found	
**miR399**	No target found	
**miR408**	Cucsa.077170.1, Cucsa.033180.1, Cucsa.049510.1	Cooper ion binding (Plantacyanin)
	Cucsa.271070.1	P1b-ATPase
**Non-conserved**		
**miR170**	No target found	
**miR827**	No target found	
**miR858**	Cucsa.096800.1, Cucsa.365190.1, Cucsa.340790.2, Cucsa.107730.1, Cucsa.107740.2, Cucsa.006170.1	R2R3 MYB transcription factor
**miR894**	Cucsa.302080.1	Acid invertase
	Cucsa.103010.1	RNA binding protein
	Cucsa.195680.2	Chloroplast protease
**miR1030**	Cucsa.254940.1	TZP; DNA binding/nucleic acid binding
	Cucsa.103950.2, Cucsa.034730.1	Chromosome condensation protein-like
	Cucsa.298340.1	Trypsin inhibitor 5
**miR2950**	Cucsa.010860.2	APK1A
	Cucsa.022840.1	Acetolactate synthase
	Cucsa.253640.1	DEAD box RNA helicase
	Cucsa.271250.1	F-box family protein
	Cucsa.097890.1	WRKY transcription factor
	Cucsa.310310.1	Clathrin assembly protein

## Discussion

Micro RNAs are key components of most of the regulatory events mediated by RNA silencing in animal and plants [23]–[25]. Although the development of high-throughput sequencing methods have contributed to detecting new as well as evolutionary conserved miRNAs [26], most of the species-specific miRNAs that frequently have low or tissue specific expression levels remain yet unidentified. By taking advantage of the recent completion of the sequence of the cucumber genome, the aim of this work was to identify the evolutionary conserved and new cucumber-specific miRNAs present in a *C. sativus* sRNAs population recovered from a cDNA library of cucumber sRNAs [17]. Our data indicated that cucumber sRNA of 24 nt dominated the pool of unique species as observed for many other herbaceous species such as *A. thaliana*, *M. truncatula* and *S. licopersicum* where 24 nts length were the most represented sRNAs [6]–[9], [19]. This high proportion of 24-nt class sRNAs in annual plants could be consequence of an active state of heterochromatin transcriptional silencing (mediated by 24-nt sRNAs) rapidly established at each generation [10]. It has been hypothesized that MIR genes originate by gene duplication events followed by random mutation processes to evolve in multiples imperfectly paired hairpins [5], [27]. Consequently, ancient evolutionary conserved miRNAs are represented by multiple MIR genes whereas non-conserved miRNAs (believed to be evolutionary recent) are generally originated from a single locus. The search for known miRNAs in cucumber has revealed a total of 25 (19 conserved and 6 non-conserved) miRNA families homologous to miRNAs sequences present on miRBase. Similarly to the results reported in other deep sequencing studies [9]–[11] diverse conserved miRNAs were not found in cucumber sRNAs data set. This situation could be explained keeping in mind that some of the conserved miRNAs reported to date are tissue-specific or accumulated in response to stress and thus, not necessarily represented in our leaf library. As expected, the great majority of the evolutionary conserved miRNAs were represented by more than one member, whereas non-conserved miRNAs identified in this study were represented by a single MIR gene.

In addition, three new cucumber miRNAs (csa-miRNAs), supported by their miRNA* strands have been identified in our *C. sativus* sRNA libraries. Another four miRNAs remain as plausible candidates as their antisense sequences could not be isolated. Moreover, 5 of these csa-miRNAs were validated by northern blot detection, which is an important requisite for the identification of new miRNAs. Based on BLASTn search against the cucumber genome and hairpin structure prediction, we identified genomic sources of miRNA and potential precursors for the totality of the new and candidate csa-miRNAs. These miRNAs are potentially generated from 7 loci and, as commonly observed in other plants, do not form transcriptional clusters. Since these miRNAs were not similar to any known miRNAs, they might be involved in more specific processes in cucumber. Unfortunately, we were not able to determine their DCL1 dependence because cucumber *dcl1* mutants are currently not available.

The majority of the new cucumber miRNAs identified here showed the canonical size expected for sRNAs derived from DCL1 processing, although sequence variants that possessed shortened or lengthened 5′ or 3′ ends were also found. For instance, csa-miR3 and csa-miR4 were 21-nt long, consistent with canonical DCL1 products, whereas csa-miR2 (20 nt) and csa-miR5 (22 nt) showed a very little size variation, perhaps due to inaccuracy of DCL1 processing [7]. Only two cucumber miRNAs (cas-miR1 and cas-miR6 - 24 nt) showed unexpected sizes. This situation could be similar to that previously observed in *Arabidopsis*, where diverse miRNA families are also independently processed by DCL3 to generate a new class of *bona fide* (23–25 nt) miRNAs with no canonical size, called long miRNAs [28]. Additionally, it was also reported that the accumulation of long miRNAs in *A. thaliana* was inversely proportional to the level of miRNA conservation and exhibit organ-specific expression patterns [28]. A similar situation was recently reported for miRNAs recovered from different grapevine tissues [10]. Future studies are necessary to determine if alternative and tissue-specific DCL processing of miRNA precursors also occur during *C. sativus* miRNA biogenesis.

Species-specific miRNAs are believed to be recently evolved and, in general, expressed at levels lower than those of strictly conserved miRNAs [5]–[7], [10], [29]. Our quantitative data obtained from sequencing frequencies of conserved and non-conserved miRNAs accommodates well to this prediction, and indicates that new cucumber miRNAs exhibit residual accumulation in the tissue tested. In comparison to recent reports in other plant species such as grapevine [10], Populus [30] or Medicago [9] the number the potential specie-specific miRNAs found in cucumber (3 specific miRNAs with *miRNA sequence) was considerably low. Although this observation could be used as a preliminary evidence that in cucumber the regulatory network mediated by specific miRNAs could be less complex that in other species, the tissue-specific nature of our library (leaves of adult plants) can again explain this low number.

Potential targets, with a wide variety of predicted functions, were identified for 4 potential new cucumber miRNAs. In concordance with previous reports most of the targets found in the cucumber transcriptome were plant specific factors related with the transcription machinery, such as the members of the MYB family (*r2r3-myb*) or regulatory proteins involved in protein-protein and/or protein-RNA interactions. The biological importance of the potential regulation of these cucumber genes by miRNAs needs further studies. Surprisingly, for 3 new miRNAs (csa-miR1, csa-miR3 and csa-miR6) predicted targets in the *C. sativus* transcriptome could not be found. However, homologous regions, candidates to be potential targets for csa-Mir1 and csa-Mir6 were identified in non-coding genomic-regions. This observation opened the possibility that these specific miRNAs could regulate the expression of cucumber non-coding RNAs, and consequently be involved in a more complex regulatory pathways related to epigenetic processes [31]. Nevertheless, the possibility that the target regions found in the recently sequenced cucumber genome are not yet correctly annotated or that the cucumber transcript dataset was still incomplete can not be ruled out. Future studies are necessary to elucidate the functional importance of these csa-miRNAs. Finally, the highly restrictive algorithms used in this approaches [32] could fail to identify the potential targets for some miRNAs (maybe because the existence of mismatches between the miRNA and their target), resembling the recently observed for the miR398 in Arabidopsis [33].

In summary, in this study we have identified, for the first time, conserved, known non-conserved and new miRNAs in cucumber, using a combined approach of high throughout sequencing and bioinformatics prediction of MIR precursors. In addition, a significant number of these miRNAs were validated by northern blot hybridization fulfilling the requirements necessary to the annotation of new plant-miRNAs [12], [13].

## Materials and Methods

### Small RNA library information

The sequences used in this work were obtained from a library generated starting from a sRNAs population recovered from leaves and phloem exudate of healthy and *Hop stunt viroid*-infected cucumber (*C. sativus*) plants and sequenced by 454 Life Science Technology (Lifesequencing, Branfor, CT, USA; www.lifesequencing.com) ([17]-NCBI/SRA accession code SRP001408).

### Bioinformatics analysis of sRNA sequences and targets prediction

Adapter trimming and cleaning of the 454 reads obtained from leaves of non-infected cucumber plants were performed by Perl scripts locally developed by the Bioinformatics Service at the Instituto de Biología Molecular y Celular de Plantas (IBMCP), Valencia, Spain (http://www.ibmcp.upv.es). Next, rRNA, tRNA, snRNA and snoRNA derived sequences were removed from our sRNAs dataset; the remaining sequences were used in this study. The unique sRNA sequences were employed to analyze the sRNAs accumulation profiles and to identify in the miRNA database (miRBase 13.0) known (conserved and non-conserved) miRNAs in cucumber. The sequences ranging the canonical miRNA length ±2 nt were considered to be known miRNAs.

To study potential precursors of new cucumber miRNAs, the sRNA sequences recovered from our library were aligned with the *C. sativus* transcript database (http://www.phytozome.net/cucumber.php) and then were processed by miRCat from the small RNA toolkit from the University of East Anglia (http://srna-tools.cmp.uea.ac.uk/plant/cgi-bin/srna-tools.cgi). The resulting structures, with minimal matched nucleotide pairs of miRNA and miRNA* exceeding 16 nt and with maximal size differences of miRNA and miRNA* up to 4 nt, were retained as new miRNA candidates. The sequence of the targets from *C. sativus* were searched against the protein database of the NCBI (National Center for Biotechnology Information, http://www.ncbi.nlm.nih.gov/) using the blastx (translated nucleotide search against protein database) option of the blast search.

### RNA isolation and Northern blot assays

Cucumber plants were maintained in environmentally controlled growing chambers at two different temperatures 30°C and 25°C, resembling the growing conditions used for sRNAs library generation [17]. Total RNA was extracted from leaves using TRI reagent (Sigma, St. Louis, MO, USA) according to the manufacturer's instructions. The low-molecular-weight RNA fraction was enriched using the miRNA Isolation Kit MIRACLE (Stratagene, La Jolla, CA, USA). Low molecular-weight enriched-RNA was loaded onto 20% polyacrylamide gels with 0.25× TBE and 8 M urea. RNA was transferred to a nylon membrane (Roche Diagnostics GmbH). Hybridization using different digoxigenin-labelled RNA as a probe was performed as described previously [34].

## Supporting Information

Figure S1 **Northern blot detection of miR159, miR167 and miR168 in cucumber plants.** The leaves were collected weekly from 4 (one group for week) independent groups of 3 different plants and pooled, before the RNA extraction. The samples were recovered at 5, 6, 7 and 8 weeks after germination (lanes 1 to 4, respectively). The 3 different plant sectors analyzed (apex, young leaves and old leaves) correspond to: first leaves and apical meristem (apex), third leaves from the shoot apex (young leaves) and fifth leaves from the shoot apex (old leaves).
(JPG)

Table S1 **Conserved and known but non-conserved miRNA families predicted by miRCat in cucumber transcriptome.**
(DOC)
