# Supplementary figures and images for: High-Throughput Sequencing, Characterization and Detection of New and Conserved Cucumber miRNAs
Source: PLoS One. 2011 May 16;6(5):e19523. doi: 10.1371/journal.pone.0019523 (PMC3095615; doi:10.1371/journal.pone.0019523)

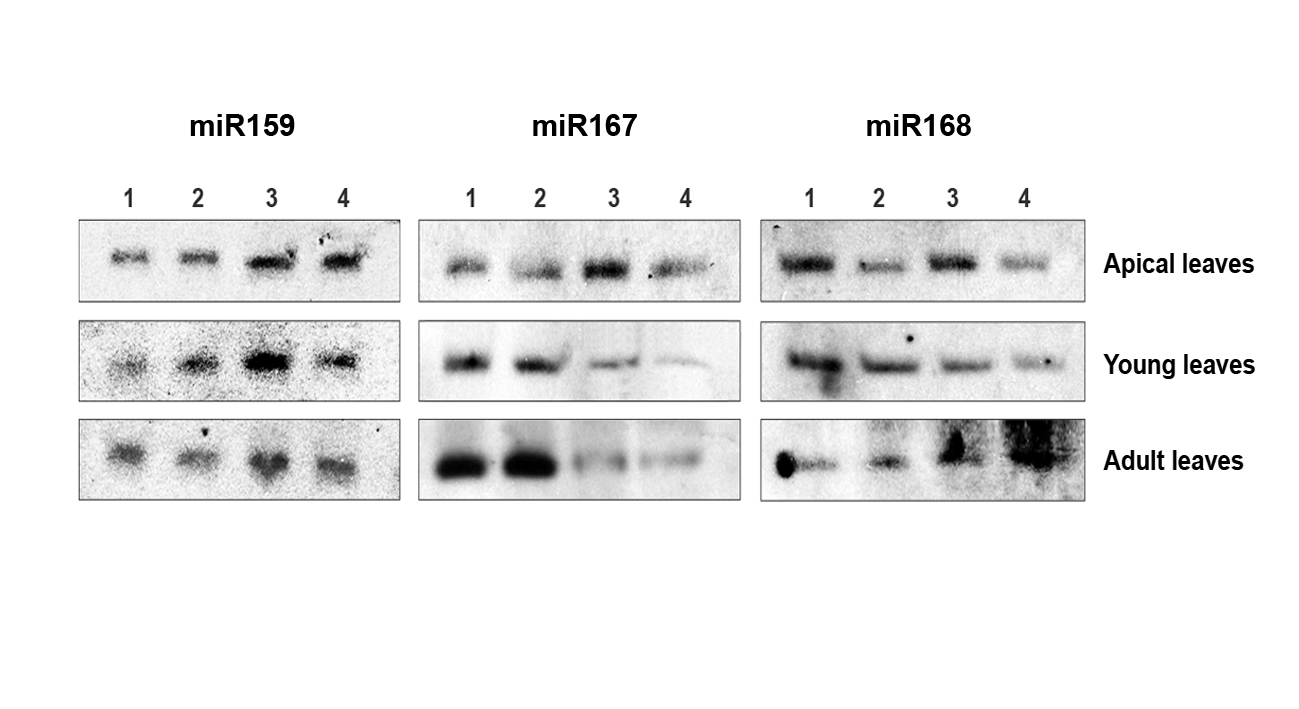

Supplement: Figure S1 — Northern blot detection of miR159, miR167 and miR168 in cucumber plants. The leaves were collected weekly from 4 (one group for week) independent groups of 3 different plants and pooled, before the RNA extraction. The samples were recovered at 5, 6, 7 and 8 weeks after germination (lanes 1 to 4, respectively). The 3 different plant sectors analyzed (apex, young leaves and old leaves) correspond to: first leaves and apical meristem (apex), third leaves from the shoot apex (young leaves) and fifth leaves from the shoot apex (old leaves). (JPG) [file pone.0019523.s001.jpg]
