# Supplementary material for: High-Throughput Sequencing, Characterization and Detection of New and Conserved Cucumber miRNAs
Source: PLoS One. 2011 May 16;6(5):e19523. doi: 10.1371/journal.pone.0019523 (PMC3095615; doi:10.1371/journal.pone.0019523)
Supplement: Table S1 — Conserved and known but non-conserved miRNA families predicted by miRCat in cucumber transcriptome. (DOC) [file pone.0019523.s002.doc]

**Supporting Table 1. Know conserved and non-conserevd miRNA families predicted by miRCat.**

| **Family** | **M** | **Msmt** | **miRNA/**  **miRNA*** | **Location** | **Orientation** | **Reads** | **Sequence** | **Length** | **Hairpin Length** | **Minimum**  **Free**  **Energy** | **Class** | **miRNA*** |
| --- | --- | --- | --- | --- | --- | --- | --- | --- | --- | --- | --- | --- |
| miR156 | a | 0 | miRNA | scaffold01037:688952_688971 | + | 10 | TGACAGAAGAGAGTGAGCAC | 20 | 164 | -59.01 | B | NO |
|  | b | 0 | miRNA | scaffold02920:91547_91566 | + | 10 | TGACAGAAGAGAGTGAGCAC | 20 | 133 | -71.30 | B | NO |
|  | c | 0 | miRNA | scaffold03504:382309_382328 | + | 10 | TGACAGAAGAGAGTGAGCAC | 20 | 237 | -100.91 | B | NO |
|  | d | 1 | miRNA | scaffold01144:2326074_2326094 | + | 37 | TTGACAGAAGAGAGTGAGCAC | 21 | 151 | -51.90 | B | NO |
|  | e | 1 | miRNA | scaffold02500:429183_429203 | - | 37 | TTGACAGAAGAGAGTGAGCAC | 21 | 138 | -68.90 | B | NO |
|  | f | 0 | miRNA | scaffold01139:565175_565195 | + | 202 | TTGACAGAAGATAGAGAGCAC | 21 | 138 | -61.80 | B | NO |
|  | g | 0 | miRNA | scaffold03569:4498_4517 | - | 1 | TGACAGAAGATAGAGAGCAC | 20 | 140 | -55.05 | C2 | NO |
| miR160 | a | 0 | miRNA | scaffold00055:628909_628929 | + | 34 | TGCCTGGCTCCCTGTATGCCA | 21 | 148 | -60.00 | B | NO |
|  | b | 0 | miRNA | scaffold01001:257130_257150 | - | 34 | TGCCTGGCTCCCTGTATGCCA | 21 | 85 | -47.10 | B | NO |
|  | c | 0 | miRNA | scaffold02978:457845_457865 | - | 34 | TGCCTGGCTCCCTGTATGCCA | 21 | 133 | -68.00 | A | GCGTATGAGGAGCCATGCATA(1) |
| miR164 | a | 0 | miRNA | scaffold01225:909647_909667 | + | 14 | TGGAGAAGCAGGGCACGTGCA | 21 | 160 | -76.10 | B | NO |
|  | b | 0 | miRNA | scaffold01251:107171_107191 | - | 14 | TGGAGAAGCAGGGCACGTGCA | 21 | 157 | -56.75 | B | NO |
|  | c | 0 | miRNA | scaffold01416:586832_586852 | + | 14 | TGGAGAAGCAGGGCACGTGCA | 21 | 100 | -50.01 | B | NO |
| miR166 | a | 0 | miRNA | scaffold01053:90145_90165 | - | 10 | TCGGACCAGGCTTCATTCCCC | 21 | 120 | -52.40 | A | GGAATGTTGTCTGGTGCGAGA(1) |
|  | b | 1 | miRNA | scaffold03882:938939_938959 | + | 1 | TCGGACCAGGCTTCATTCTCG | 21 | 153 | -53.90 | C2 | NO |
|  | c | 0 | miRNA* | scaffold00998:2985377_2985397 | - | 154 | GGAATGTTGTCTGGCTCGAGG | 21 | 169 | -52.90 | A | TCGGACCAGGCTTCATTCCCC(10) |
|  | d | 0 | miRNA* | scaffold02229:6002600_6002620 | + | 154 | GGAATGTTGTCTGGCTCGAGG | 21 | 95 | -44.14 | A | TCGGACCAGGCTTCATTCCCC(10) |
|  | e | 0 | miRNA* | scaffold02951:1938556_1938576 | - | 154 | GGAATGTTGTCTGGCTCGAGG | 21 | 186 | -59.41 | A | TCGGACCAGGCTTCATTCCCC(10) |
|  | f | 0 | miRNA* | scaffold01274:414228_414248 | + | 2 | GGAATGTTGTTTGGCTCGAGG | 21 | 163 | -63.30 | A | TCTCGGACCAGGCTTCATTCC(1) |
| miR167 | a | 0 | miRNA | scaffold00542:3510078_3510098 | + | 51 | TGAAGCTGCCAGCATGATCTG | 21 | 121 | -54.80 | B | NO |
|  | b | 0 | miRNA | scaffold02310:29259_29279 | + | 51 | TGAAGCTGCCAGCATGATCTG | 21 | 123 | -55.00 | A | GATCATATTGGTAGCTTCATC(9) |
|  | c | 0 | miRNA | scaffold00227:154472_154492 | - | 8 | TGAAGCTGCCAGCATGATCTA | 21 | 127 | -46.20 | B | NO |
|  | d | 0 | miRNA | scaffold02310:19947_19967 | + | 8 | TGAAGCTGCCAGCATGATCTA | 21 | 170 | -57.60 | B | NO |
|  | e | 1 | miRNA | scaffold03918:322742_322763 | - | 31 | TCAAGCTGCCAGCATGATCTAA | 22 | 156 | -49.50 | B | NO |
| miR169 | a | 0 | miRNA | scaffold00894:379625_379645 | + | 3 | AAGCCAAGGATGAATTGCCGG | 21 | 197 | -77.90 | A | GGCAATTCCATTCTTGGCTAA(1) |
|  | b | 1 | miRNA | scaffold01153:1413276_1413297 | + | 4 | TAGCCAAAGATGACTTGCCTGT | 22 | 195 | -79.90 | C1 | NO |
|  | c | 1 | miRNA | scaffold01153:1417797_1417818 | + | 4 | TAGCCAAAGATGACTTGCCTGT | 22 | 196 | -76.39 | C1 | NO |
|  | d | 2 | miRNA | scaffold00817:120051_120073 | + | 1 | TGAGCCAAGAATGACTTGCCGGC | 23 | 175 | -80.11 | C2 | NO |
|  | e | 0 | miRNA* | scaffold00923:21016_21035 | - | 2 | GCAAGTCGTCCTTGGCTACC | 20 | 164 | -81.30 | C1 | NO |
| miR171 | a | 0 | miRNA | scaffold01107:639271_639291 | - | 40 | TGATTGAGCCGTGCCAATATC | 21 | 194 | -65.55 | B | NO |
|  | b | 0 | miRNA | scaffold01141:244487_244507 | - | 40 | TGATTGAGCCGTGCCAATATC | 21 | 98 | -41.60 | A | TGTTGGAACGGTTCAATCAAA(1) |
|  | c | 0 | miRNA | scaffold03356:5524104_5524124 | - | 40 | TGATTGAGCCGTGCCAATATC | 21 | 184 | -51.99 | A | TATTGGCCCGGTTCACTCAGA(1) |
|  | d | 0 | miRNA | scaffold00464:182497_182517 | - | 21 | TGATTGAGCCGCGCCAATATC | 21 | 145 | -57.30 | B | NO |
|  | e | 0 | miRNA | scaffold01037:317696_317716 | + | 21 | TGATTGAGCCGCGCCAATATC | 21 | 160 | -51.14 | B | NO |
|  | f | 0 | miRNA | scaffold04100:161935_161955 | - | 21 | TGATTGAGCCGCGCCAATATC | 21 | 145 | -43.80 | B | NO |
|  | g | 0 | miRNA | scaffold00765:155091_155111 | + | 2 | TTGAGCCGTGCCAATATCACG | 21 | 148 | -44.27 | C1 | NO |
|  | h | 1 | miRNA | scaffold00765:133580_133600 | - | 3 | TTGAGCCGCGTCAATATCTCT | 21 | 141 | -45.90 | C1 | NO |
| miR172 | a | 0 | miRNA | scaffold01053:287019_287039 | + | 35 | AGAATCTTGATGATGCTGCAT | 21 | 158 | -54.00 | A | GCGGCATCATCAAGATTCACA(4) |
|  | b | 0 | miRNA | scaffold03904:1097597_1097617 | + | 35 | AGAATCTTGATGATGCTGCAT | 21 | 239 | -87.20 | A | GTAGCATTATCAAGATTCACA(7) |
|  | c | 0 | miRNA | scaffold01066:88458_88477 | + | 4 | GAATCTTGATGATGCTGCAT | 20 | 190 | -58.91 | C1 | NO |
|  | d | 0 | miRNA | scaffold00154:286749_286769 | + | 2 | GAGAATCTTGATGATGCTGCA | 21 | 193 | -60.31 | C1 | NO |
| miR319 |  | 0 | miRNA | scaffold02653:291888_291908 | - | 2 | TTGGACTGAAGGGAGCTCCCT | 21 | 216 | -91.66 | C1 | NO |
| miR390 | a | 0 | miRNA | scaffold01029:141650_141670 | + | 18 | AAGCTCAGGAGGGATAGCGCC | 21 | 126 | -55.90 | A | CGCTATCCATCCTGAGTTTCC(5) |
|  | b | 0 | miRNA | scaffold01066:712440_712460 | + | 18 | AAGCTCAGGAGGGATAGCGCC | 21 | 130 | -50.40 | B | NO |
|  | c | 0 | miRNA | scaffold01119:204265_204285 | + | 18 | AAGCTCAGGAGGGATAGCGCC | 21 | 146 | -57.10 | B | NO |
|  | d | 0 | miRNA | scaffold01225:840228_840248 | + | 18 | AAGCTCAGGAGGGATAGCGCC | 21 | 150 | -74.10 | B | NO |
| miR393 | a | 0 | miRNA | scaffold00931:100515_100536 | - | 3 | TCCAAAGGGATCGCATTGATCC | 22 | 161 | -60.90 | C1 | NO |
|  | b | 0 | miRNA | scaffold01044:1042192_1042213 | + | 3 | TCCAAAGGGATCGCATTGATCC | 22 | 160 | -48.70 | C1 | NO |
|  | c | 0 | miRNA | scaffold01153:124459_124480 | + | 3 | TCCAAAGGGATCGCATTGATCC | 22 | 116 | -38.90 | C1 | NO |
| miR396 | a | 0 | miRNA | scaffold00464:137922_137942 | - | 3 | TTCCACAGCTTTCTTGAACTT | 21 | 283 | -96.30 | C1 | NO |
|  | b | 0 | miRNA | scaffold00464:141897_141917 | - | 3 | TTCCACAGCTTTCTTGAACTT | 21 | 151 | -37.76 | C1 | NO |
|  | C | 0 | miRNA | scaffold01037:350259_350279 | + | 3 | TTCCACAGCTTTCTTGAACTT | 21 | 173 | -64.40 | A | GTTCAAGAAAGCTGTGGGAGA(2) |
|  | d | 0 | miRNA* | scaffold00464:132023_132043 | + | 12 | GTTCAATAAAGCTGTGGGAAG | 21 | 198 | -69.76 | A | TTCCACAGCTTTCTTGAACTG(3) |
|  | e | 0 | miRNA* | scaffold01037:357863_357883 | - | 12 | GTTCAATAAAGCTGTGGGAAG | 21 | 174 | -57.06 | A | TTCCACGGCTTTCTTGAACTG(3) |
| miR397 |  | 0 | miRNA | scaffold01017:160952_160972 | + | 9 | CATTGAGTGCAGCGTTGATGT | 21 | 132 | -40.75 | B | NO |
| miR398 |  | 1 | miRNA | scaffold02653:1332389_1332409 | + | 1 | TTGTGTTCTCAGGTCACCCCT | 21 | 175 | -60.20 | C2 | NO |
| miR399 | a | 2 | miRNA | scaffold00542:2253810_2253830 | + | 1 | TGCCAAAAGAGACTTGCCCTG | 21 | 149 | -46.63 | C2 | NO |
|  | b | 2 | miRNA | scaffold00542:2256909_2256929 | + | 1 | TGCCAAAAGAGACTTGCCCTG | 21 | 101 | -47.10 | C2 | NO |
| miR408 |  | 0 | miRNA | scaffold00789:610115_610136 | - | 5 | TGCACTGCCTCTTCCCTGGCTG | 22 | 208 | -77.02 | B | NO |
| miR827 |  | 1 | miRNA | scaffold02352:635236_635256 | - | 3 | TTAGATGACCATCAACGAACG | 21 | 130 | -41.43 | C2 | NO |
| miR2950 |  | 0 | miRNA* | scaffold03625:75025_75045 | + | 6 | TGGTGTGCATGAGATGGAATA | 21 | 107 | -64.40 | A | TTCCATCTCTTGCACACTGGA(2) |
